# Fully automated grey and white matter spinal cord segmentation

**DOI:** 10.1038/srep36151

**Published:** 2016-10-27

**Authors:** Ferran Prados, M. Jorge Cardoso, Marios C. Yiannakas, Luke R. Hoy, Elisa Tebaldi, Hugh Kearney, Martina D. Liechti, David H. Miller, Olga Ciccarelli, Claudia A. M. Gandini Wheeler-Kingshott, Sebastien Ourselin

**Affiliations:** 1 Translational Imaging Group, Centre for Medical Image Computing (CMIC), Department of Medical Physics and Bioengineering, University College London, Malet Place Engineering Building, London, WC1E 6BT, UK; 2 NMR Research Unit, Queen Square MS Centre, Department of Neuroinflammation, UCL Institute of Neurology, 1st Floor, Russell Square House, 10-12 Russell Square, London, WC1B 5EH, UK; 3 Dementia Research Centre, Department of Neurodegenerative Disease, UCL Institute of Neurology, Queen Square, London, WC1N 3BG, UK; 4 Brain MRI 3T Center, C. Mondino National Neurological Institute, Pavia, Italy

## Abstract

Axonal loss in the spinal cord is one of the main contributing factors to irreversible clinical disability in multiple sclerosis (MS). *In vivo* axonal loss can be assessed indirectly by estimating a reduction in the cervical cross-sectional area (CSA) of the spinal cord over time, which is indicative of spinal cord atrophy, and such a measure may be obtained by means of image segmentation using magnetic resonance imaging (MRI). In this work, we propose a new fully automated spinal cord segmentation technique that incorporates two different multi-atlas segmentation propagation and fusion techniques: The Optimized PatchMatch Label fusion (OPAL) algorithm for localising and approximately segmenting the spinal cord, and the Similarity and Truth Estimation for Propagated Segmentations (STEPS) algorithm for segmenting white and grey matter simultaneously. In a retrospective analysis of MRI data, the proposed method facilitated CSA measurements with accuracy equivalent to the inter-rater variability, with a Dice score (DSC) of 0.967 at C2/C3 level. The segmentation performance for grey matter at C2/C3 level was close to inter-rater variability, reaching an accuracy (DSC) of 0.826 for healthy subjects and 0.835 people with clinically isolated syndrome MS.

Histopathological studies have provided evidence of spinal cord grey matter (GM) and white matter (WM) changes in a large spectrum of neurological conditions^1^. In particular, the loss of axons in the spinal cord is thought to represent one of the main contributing factors to clinical disability. *In vivo*, axonal loss can be assessed indirectly by estimating the reduction in cervical cord cross-sectional area (CSA) over time (i.e., spinal cord atrophy) from magnetic resonance imaging (MRI) through the use of image segmentation methods; the reduction in CSA over time has been shown to correlate with clinical scores of physical disability^2^,^3^,^4^. It must be noted that the measure of cord CSA alone cannot differentiate between the individual rates of GM and WM atrophy, which may have different clinical relevance, and should be best studied independently^5^. However, in pathologies such as multiple sclerosis (MS) where the presence of focal and diffuse lesions may obscure the boundary between tissue-types^6^, tissue-specific segmentation can be challenging, and may potentially lead to biased morphometric estimates. Spinal cord GM area was the strongest correlate of disability in MS using multivariate models that include brain GM and WM volumes, fluid-attenuated inversion recovery lesion load, T1 lesion load, spinal cord CSA, number of spinal cord T2 lesions, age, sex, and disease duration^3^,^4^.

Over the last two decades, a number of semi-automated segmentation methods have been proposed for the estimation of spinal cord CSA^2^,^7^,^8^,^9^,^10^,^11^,^12^,^13^,^14^,^15^, and more recently, methods that jointly segment both CSA and GM^16^,^17^,^18^,^19^,^20^. Recent studies by El Mendili *et al*.^14^,^21^, which are based on the threshold-based method (TbM) previously described by Losseff *et al*.^2^, has shown high reproducibility and accuracy in measuring cord CSA using a semi-automated double threshold-based method (DTbM) that requires minimal manual intervention. As compared to the reference method, DTbM had a Dice Similarity Coefficient (DSC) value of 0.98 for cord CSA at C2 vertebral level in a group of 82 healthy subjects and 30 patients with various neurological conditions such as amyotrophic lateral sclerosis, spinal muscular atrophy and spinal cord injury. While semi-automated methods have improved considerably over the years, reducing intra- and inter-rater variability could have direct implications for future clinical trials of neuroprotection or longitudinal observational studies.

Multi-atlas automated segmentation methods have been used successfully in different parts of the brain. A fully automated spinal cord segmentation method, based on an iterative Non-Local Simultaneous Truth And Performance Level Estimation (*iNLS*) multi-atlas framework, has also been presented recently^16^. The method demonstrated high segmentation accuracy for the non-pathological spinal cord with a median DSC of 0.81 for CSA segmentation and 0.72 for GM segmentation. More specifically, the *iNLS* multi-atlas framework uses 3 degrees-of-freedom rigid body transform per template propagation step in order to achieve the reported accuracy.

Recently, a different multi-atlas segmentation propagation and fusion technique called STEPS (Similarity and Truth Estimation for Propagated Segmentations)^22^ has demonstrated a statistically significant increase in segmentation accuracy in several key brain structures such as the hippocampus compared with other methods. The algorithm operates in two steps as follows. In the first step, there is the segmentation propagation, where each element of the template library is registered to the target image using an affine registration (12 degrees-of-freedom), followed by a non-rigid registration; in the second step, the best deformed templates are selected based on the locally normalised cross correlation, and are fused into a consensus segmentation. The combination of a local similarity metric for image fusion and the use of non-rigid registration for template propagation makes STEPS a good candidate algorithm for CSA and GM segmentation. However, the affine/non-rigid registration steps require a good initialisation to achieve high accuracy.

Another patch-based multi-altas segmentation and fusion technique is PatchMatch^23^,^24^,^25^. While the original PatchMatch algorithm was designed to look for similarities between two 2D images^26^, multiple extensions to include 3D MR images have been proposed since. Of relevance here is the optimized PatchMatch Label fusion (OPAL), which was first used to produce accurate and fast segmentations of the hippocampus^23^,^25^. This work has been recently extended to multi-modal data (4D) for the purpose of MS lesion detection^24^. PatchMatch methods are normally good at localising and roughly segmenting objects in images due to the computational efficiency of the OPAL random search algorithm, albeit with reduced accuracy.

In this work, we propose a new fully automated spinal cord segmentation technique that incorporates two different multi-atlas segmentation propagation and fusion algorithms: OPAL and STEPS. The OPAL random search is used to localise and roughly segment the spinal cord, and the STEPS algorithm is then used to perform slice-wise tissue segmentation. The proposed method is evaluated retrospectively using a dataset that includes healthy volunteers and people with MS.

## Methods

### Segmentation methods

**Figure 1:**
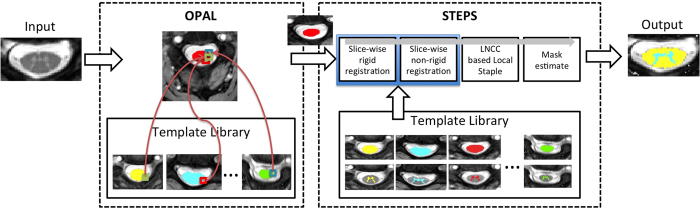
Schematic representation of the proposed pipeline. The spinal cord images were cropped only for visualisation purposes.

The proposed method combines two existing label fusion segmentation techniques: OPAL for detecting the spinal cord and STEPS for accurately segmenting the GM and whole spinal cord. Bearing in mind that the typical slice thickness of spinal cord images is approximately 3–5 mm^27^, the proposed method performs all registrations and segmentations in a 2D slice-wise manner before merging them into a 3D volume. A schematic representation of the proposed pipeline is shown in Fig. 1. Additional details pertaining to each step individually are discussed in subsequent sections.

#### Step 1: Localisation using OPAL

While the original PatchMatch algorithm was designed to match 2D patches within the same image^26^, the method has been recently extended to 3D^23^,^25^ and 4D data^24^, with the search range extended to an external reference library. Due to the excellent performance in terms of computational time and good segmentation accuracy in some applications, OPAL is used here in its original form to simply localise the spinal cord. This cord localisation step is achieved by providing an external database of spinal cord images and associated manually segmented cords to the OPAL algorithm, all of which are then propagated to the new unseen image. This step has an average computational time of less than 1 *sec*. While OPAL was found to perform well both for cord and GM segmentations in healthy volunteers, the segmentation performance degraded rapidly in the presence of pathology (e.g. MS lesions), not allowing to use the default application over MS subjects. OPAL is publicly available inside NiftySeg software package (niftyseg.sf.net).

#### Step 2: Segmentation using STEPS

The main characteristic of STEPS is that it introduces a spatially variant image similarity term in a STAPLE framework^22^, enabling the characterisation of both image similarity and human rater performance in a unified manner. The STEPS segmentation process is divided into two stages: segmentation propagation and fusion. Starting from a template library with associated manual segmentations, all the templates (excluding the image under analysis) are first registered to the target image using initially a rigid-only registration and then a non-rigid registration. All registration was done using the NiftyReg software package (niftyreg.sf.net). The normalised cross correlation (NCC) was then estimated between each deformed template and the target image, quantifying the similarity between the two images. The most similar deformed templates according to the NCC were finally fused into a consensus segmentation according to the locally normalised cross correlation (LNCC) between the registered template images and the target image. A consensus probabilistic CSA and GM segmentation is obtained using the STEPS algorithm as implemented in NiftySeg (niftyseg.sf.net). The probabilistic nature of the consensus segmentation implicitly encodes partial volume effect, improving tissue boundary localisation and delineation. Finally, to produce binary segmentations, the probabilistic consensus masks were thresholded at 0.5.

Note that, in order to increase the performance of the fusion step, the centre of mass of the OPAL cord segmentation was used to initialise the rigid registration step between templates and target image. The OPAL cord segmentation was also used to mask non-cord regions from the non-linear registration step, further improving the performance of the template registration step.

### Algorithm Parameters

The pipeline works in a slice-wise manner (2D). All experiments used the following parameters. OPAL was used with the original parameters^23^,^25^. The 2D patch size was 5 × 5 voxels and the number of inner iterations was 5. Finally, the numbers of threads and the number of best-matches were both set to 10.

The OPAL mask was then dilated eight times to ensure that there was sufficient boundary information to perform both affine and non-rigid registrations^28^,^29^.

For STEPS, the parameters used were as suggested by Cardoso *et al*.^22^. The number of best templates was *X* = 15, standard deviation of the Gaussian smoothing kernel for LNCC estimation *σ* = 1.5 and the Markov Random Field (MRF) spatial consistency set to 0.55.

The linear registration of the templates for the STEPS label fusion has been done using a symmetric 6 degree-of-freedom affine registration using a block matching approach^30^,^31^. The non-rigid alignment has been done using the fast free-form registration algorithm^32^ with the LNCC (*σ* = 1) as a similarity measure. The pyramidal approach was set to use 6 levels, and the grid spacing at the lowest level of the pyramid set to 5 voxels.

The input image was resampled to the template library resolution using a linear interpolation, and the obtained binary masks were resampled back to the original resolution using nearest-neighbour interpolation in order to maintain their categorical nature.

### Data

We analysed data acquired in 102 subjects participating in a study of spinal cord MR imaging in MS^33^. There were: 25 healthy controls, 19 people with clinically isolated syndrome (CIS), 20 with primary progressive multiple sclerosis (PPMS), 17 with secondary progressive multiple sclerosis (SPMS), and 21 with relapsing remitting multiple sclerosis (RRMS). We recruited people with different types of MS because they are expected to have a different degree of pathological changes in the spinal cord. MS lesions involving at least two spinal cord WM columns and extending to the GM are more frequent in SPMS than in the early phase of MS^33^. Written informed consent was obtained from all participants and this work was approved by the local research ethics committee.

Using a 3T Philips Achieva MRI system with dual-transmit technology (Philips Healthcare, Best, Netherlands) and the manufacturer’s product 16-channel neurovascular (NV) coil, the cervical cord was imaged in the axial-oblique plane (i.e. slices perpendicular to the cord) with the center of the imaging volume positioned at the level of C2-3 intervertebral disc. The MRI acquisition parameters were: Fat-suppressed 3*D* slab-selective fast field echo (3D-FFE) with TR = 23 ms; TE = 5 ms, flip angle *α* = 7 degrees, FOV = 240 × 180 mm, voxel size = 0.5 × 0.5 × 5 *mm*^3^, NEX = 8, 10 contiguous axial slices, scanning time = 13:34 min.

**Figure 2:**
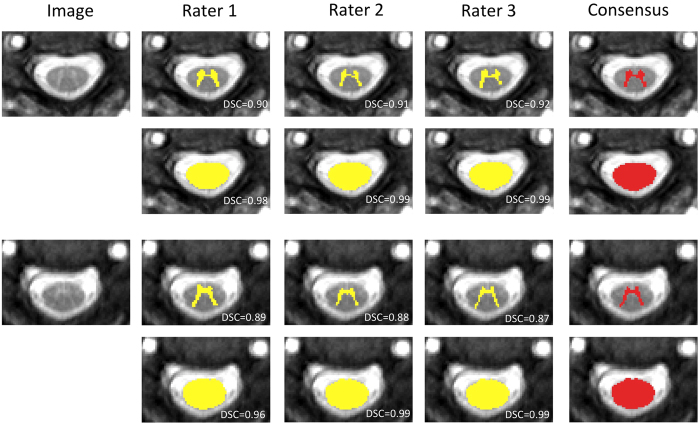
Example of GM and CSA consensus masks obtained from the three raters masks intersection. Consensus mask for a healthy control (rows 1 and 2) and a RRMS patient (rows 3 and 4). DSC respects the consensus mask is overlayed in each segmentation.

Three expert raters working independently and with the raw images, first outlined GM manually and semi-automatically outlined the cord CSA using the ‘cord finder’^10^ option available with JIM v.6 (Xinapse systems, www.xinapse.com/). Based on the previously published methodology by Losseff *et al*.^2^, a 15 mm section of the high-resolution 3D-FFE volumetric scan (i.e. 3 slices) was extracted, with the middle slice passing through the C2-3 intervertebral disc. ‘Cord finder’ option runs an active surface model that it needs the manual place of a seed. Raters manually positioned a seed in the centre of the cord in each of the three slices. For each subject, the associated consensus segmentation of the three raters (at least two raters agree) for the cord CSA and GM were estimated using majority voting, see Fig. 2.

The C2-3 cervical cord level was used to estimate the performance of the proposed algorithm as the inter-rater variability was found to be lower in cord areas surrounded by CSF. Nonetheless, the application to other spinal cord segments, such as thoracic or lumbar areas would only require an area specific template database. Losseff *et al*.^2^ indicates that, within the cord, this location (C2-3 level) yields the most reproducible cross-sectional area values and it is the most convenient region to measure atrophy in the spinal cord.

To avoid bias due to lack of consensus between manual segmentations, segmentations with a 3D DSC lower than 0.7 between two of the three expert raters, or images of poor quality (due to noise, motion, *etc*.) were excluded from this study. A total of 7 subjects (4 PPMS, 2 SPMS and 1 RRMS) were excluded, which resulted in a final group of 95 subjects (25 controls, 19 CIS, 16 PPMS, 15 SPMS and 20 RRMS). Table 1 shows the demographic information of the selected data.

### Template library

The template library used in this work consisted of 3D imaging volumes from the 95 subjects described in the Data section. For each subject there were three 2D slices, with the centre slice through the C2-3 level. In order to maximise the size of the library, all the scans were left-right flipped, resulting in a final template library of 570 2D images in total. For each image in the template library, the associated consensus segmentation of the three raters for the cord CSA and GM was used as template (see Fig. 2).

### Leave-one-out cross-validation

The proposed fully automated method, which estimates the CSA and segments the GM at the same time, was compared to the consensus segmentation of 3 raters. We also compared the performance of the proposed method to the *iNLS* multi-atlas framework - a fully automated GM/WM spinal cord segmentation method^16^. *iNLS* has been used with the parameters presented in Asman *et al*.^16^. A leave-one-out strategy was used for both *iNLS* and the proposed method, i.e. the image being segmented as well as its left-right flipped version^15^, were removed from the template library during segmentation. Both *iNLS* and the proposed method used the same library as described in the Data section.

All binary segmentations obtained using the proposed method were compared to the manual segmentations, the consensus segmentation and the iNLS results. To assess the performance, we used the 3D DSC, the mean surface distance (MD) and the Hausdorff distance (HD) between the masks^15^,^34^. Statistical differences in performance were estimated using a two tail unequal variance paired t-test as the error distribution was approximately Gaussian in most experiments. Furthermore, all patients’ data (CIS, PPMS, SPMS and RRMS) were split into two independent groups, one with visible lesions at C2/C3 level and one without, in order to assess the impact of the presence of lesions on the segmentation performance.

### Test-retest assessment

For assessing the reliability of the presented method we performed a test-retest experiment. Test-retest data were composed of 5 different healthy controls that repeated the same MR imaging protocol 3 times on separate occasions, with a minimum of a week and a maximum of two weeks between measurements. We segmented the images using the same library as described in Data Section and one rater analysed all the data.

## Results

**Figure 3:**
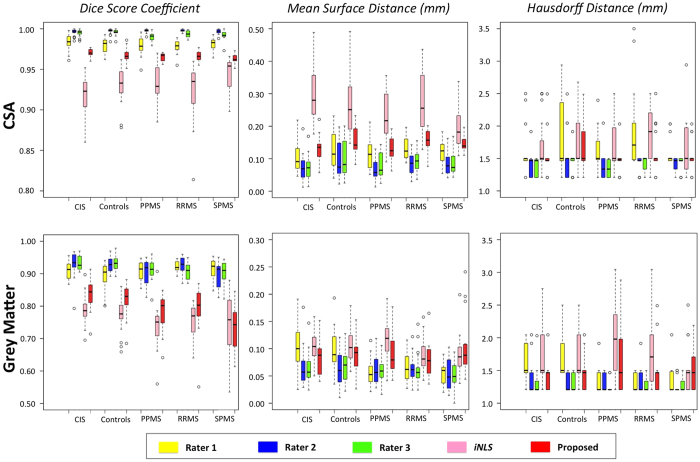
Quantitative analysis per patient group on GM and CSA segmentation of the spinal cord.

**Figure 4:**
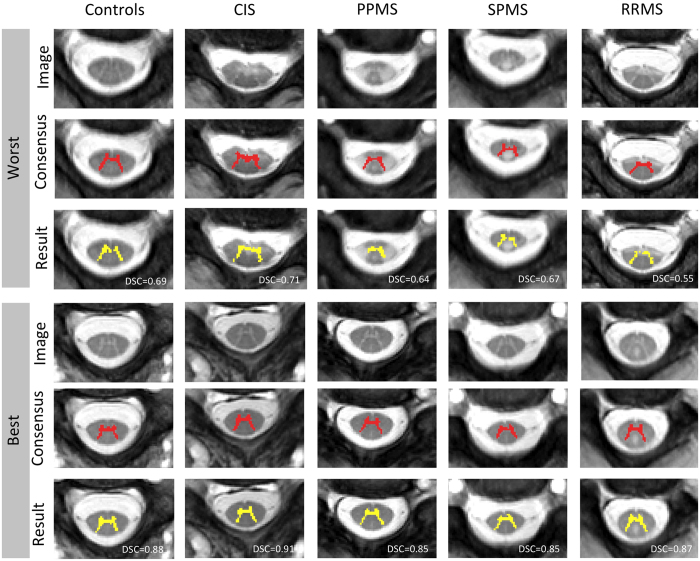
GM segmentation results showing the worst (1–3) and the best (4–6) result for each patient group. Rows 1 and 4 show one slice of the input image, rows 2 and 5 correspond to the consensus segmentation, and rows 3 and 6 are the obtained segmentation by the proposed method with DSC overlay.

Tables 2, 3 and Fig. 3, show the evaluation results for the CSA and GM segmentation at C2/C3 level respectively, with the mean (std) and p-value for the DSC, MSD and HD estimated with respect to the consensus masks. In Tables 2 and 3, significant differences (*p* < 0.05) have been marked with “*”. For the qualitative results, the best and the worst DSC results are shown for each patient group for GM segmentation (see Fig. 4).

We have found that the proposed method provides more accurate results for the CSA segmentation at C2/C3 level when compared to *iNLS* (DSC and MSD - *p* < 0.05) for all groups. When comparing the proposed method (see Table 3) with the *iNLS*, superior results were found for healthy controls, however, lower performance, but not statistically significant, is obtained for GM DSC on SPMS group. There are significant differences for GM segmentation (DSC - *p* > 0.05) between the methods for CIS, PPMS and RRMS subjects. Overall, the proposed method outperforms the state-of-the-art method *iNLS* (*p* < 0.05) for CSA and GM segmentation at C2/C3 level (see last row in Tables 2 and 3), and for GM segmentation the obtained MSD and HD by Rater 1 are not significantly different from proposed method.

Table 4 shows the results from splitting the patients’ data into two groups depending on the presence of visible lesions at the C2/C3 level. There were significant differences for CSA and GM segmentation between the absence and presence of lesions. Finally, Table 5 presents the reliability assessment results for CSA and GM regions mean and std.

Experiments were performed on a server (12 CPUs 2.8 Ghz and 64 Gb RAM). The average computational time was 30 minutes per target image using only 2 CPU threads.

### Spinal Cord GM Challenge 2016

The proposed method was used to segment 40 healthy spinal cord images from 4 independent centres and 2 different vendors as part of a spinal cord segmentation challenge that was organized as a satellite of ISMRM 2016 (cmictig.cs.ucl.ac.uk/niftyweb/program.php?p=CHALLENGE). The challenge was comprised of 6 teams with widely varying approaches ranging from multi-atlas propagation^20^ and deep learning^35^, to active contours^19^ and variational Bayes probabilistic segmentation^17^. The proposed method (named as JCSCS in the challenge) obtained the highest rank in 5 out of 10 evaluation metrics, with a mean(std) DSC, MSD, HD of 0.79(0.04), 0.39(0.44), 2.65(3.34) respectively. A future publication presenting an extended and more detailed analysis of the results is currently under preparation.

## Discussion

We propose the combined use of OPAL and STEPS for the segmentation of the whole spinal cord CSA and GM. Although we have found the output of OPAL was highly dependent on the amount of noise and the presence of lesions (producing highly spurious segmentations in some situations), our results nonetheless demonstrate that OPAL can provide a sufficiently accurate initial segmentation of the spinal cord in order to facilitate a constrained STEPS label fusion process.

Asman *et al*.^16^,^36^ highlight some caveats of using non-rigid registration for spinal cord imaging. In the proposed method, the OPAL segmentation was used to initialise and constrain, providing a region mask to the slice-wise non-rigid registration performed by STEPS, thus ameliorating most of these concerns. The computational time needed to perform these slice wise non-rigid registrations was also deemed adequate, taking approximately one second per slice and template. Moreover, the use of non-rigid registration reduces the need for a refinement step as proposed in Asman *et al*.^36^.

The fact that the proposed method gets lower, but not statistically different, performance for GM DSC on SPMS group (see Table 3) maybe due to the low number of subjects and the presence of large lesions. The use of non-rigid registration in the proposed method might be detrimental in subjects with very large lesions, as the non-rigid step introduces too much flexibility and uncertainty when deforming the candidate labels (see columns SPMS and RRMS at Fig. 4). Overall, similar differences were found for the MSD and HD scores. Table 4 shows the effect of the presence or absence of lesions, these differences are specially large and significant for GM (DSC and MSD *p* < 0.05).

Results in Tables 2 and 3 show that the proposed method is able to segment the CSA with a very high accuracy, and only slightly different to the three rater consensuses. Method gets a DSC of 0.968 that is not a clinically significant difference. The low variability between raters for CSA segmentation is thanks to the semi-automated nature of the ‘cord finder’ method from JIM. We would like to note that the results presented have been obtained in a three slice subset of the full volume. As spinal cord imaging usually has dozens or hundreds slices per volume, consistency across slices can be exploited in the future to reduce the amount of errors produced by the proposed algorithm. Moreover, the reduced number of slices and voxels per mask, in conjunction with the evaluation metrics, highlight the quality of the obtained results. Covering a larger spinal cord area would hide any small miss-segmented area due to repetitive spinal cord nature.

On a group specific basis, the CSA segmentation performed very well in all the groups and the presence of lesions has a low impact (see Table 4). Test-retest assessment also confirms these results (see Table 5). It is also important to note that the proposed method has a lower CSA segmentation HD than Rater 1 for all groups, suggesting that the errors introduced by human raters with different levels of expertise or training can be larger than the errors introduced by the proposed method, further justifying the need for advanced automated segmentation techniques for multi-centre studies and trials.

Regarding the GM segmentation, the proposed method provided acceptable results for the groups without lesions (healthy controls and CIS), achieving better MSD and HD than Rater 1. While Rater 1 tended to over-segment the GM when comparing to the consensus mask, the presented method had a tendency to only slightly under-segment the GM when compared to the consensus mask, resulting in lower MSD and HD. On a group specific basis, the GM segmentation performed better in healthy controls and CIS patients than in the other three MS groups, which is likely to be related to the limited number of lesions identified in the CIS cohort^33^. GM test-retest assessment results are also promising (see Table 5).

From Figs 3 and 4, one can observe that the worst results correspond to outliers in terms of Dice score. In these situations, the presence of hyperintensities favour the use of anatomical *a priori* assumptions by the human raters. In future we will include anatomical priors and uncertainty estimates in the segmentation process so that the segmentation algorithm reverts back to the prior model in very uncerta*in situ*ations.

The state-of-the-art results obtained as part of the ISMRM 2016 satellite spinal cord challenge further demonstrate that the proposed method is robust to differences in scanner manufacturer and acquisition parameters when compared to 4 independent rater segmentations.

In summary, this paper introduces a fully-automated GM and whole spinal cord segmentation technique based on a collaborative effort between two multi-label fusion techniques. OPAL quickly finds the cord and then STEPS segments GM and CSA. Both healthy and different types of MS subjects were used for performance assessment, and the results have been compared to the consensus of three expert manual segmentations. The accuracy of the proposed method was found to be close, comparable and in some situations better, than a single rater. GM segmentation results obtained in PPMS, SPMS and RRMS subjects showed promising results but further work is necessary to remove the influence of MS lesions.

The proposed method has only been applied to the cervical area (C2-3 level) of the spinal cord because it is the most convenient region to measure atrophy in the spinal cord in MS. Specifically, the obtained results for the CSA segmentation suggests that it may be used in a large multi-centre neuroprotective trials in progressive MS. Moreover, application to other spinal cord segments, such as thoracic or lumbar areas, or other sequences would only require an area/sequence specific template database.

## Additional Information

**How to cite this article**: Prados, F. *et al*. Fully automated grey and white matter spinal cord segmentation. *Sci. Rep*. **6**, 36151; doi: 10.1038/srep36151 (2016).

**Publisher’s note:** Springer Nature remains neutral with regard to jurisdictional claims in published maps and institutional affiliations.

## Figures and Tables

**Table 1:** Demographic data for each group.

	Control (N = 25)	CIS (N = 19)	PPMS (N = 16)	SPMS (N = 15)	RRMS (N = 20)
**Gender**	7F:18M	7F:12M	9F:7M	5F:10M	8F:12M
**Age***Mean* (*std*)	40.4(10.1)	36.0(4.6)	52.5(11.0)	55.2(7.4)	41.8(9.1)
**Disease duration***Mean* (*std*)	—	0.4(0.3)	11.5(8.7)	22.1(14.1)	7.8(6.1)
**EDSS score***Median* (*range*)	—	1(0–3.5)	6(2–7)	6.5(4.5–7.5)	2.5(0–6.5)

First row: gender - female(F):male(M); second row: mean age in years; third row: mean disease duration in years; and last row: median EDSS score. Std: standard deviation.

**Table 2:** Evaluation results for the CSA segmentation, with the mean (std) Dice score coefficient (DSC), mean surface distance (MSD), Hausdorff distance (HD) (all with respect to the consensus).

		Rater 1	Rater 2	Rater 3	*iNLS*	Proposed
*Controls* (*N* = *25*)	*DSC*	0.980 (0.009)*	0.997 (0.004)*	0.996 (0.003)*	0.931 (0.021)*	0.968 (0.008)
	*MSD*	0.130 (0.056)*	0.096 (0.056)*	0.101 (0.052)*	0.265 (0.094)*	0.153 (0.042)
	*HD*	1.785 (0.544)	1.494 (0.302)*	1.448 (0.150)*	1.809 (0.429)	1.649 (0.349)
*CIS* (*N* = *19*)	*DSC*	0.983 (0.010)*	0.996 (0.004)*	0.995 (0.004)*	0.917 (0.025)*	0.971 (0.005)
	*MSD*	0.107 (0.051)*	0.071 (0.043)*	0.072 (0.036)*	0.295 (0.088)*	0.132 (0.037)
	*HD*	1.598 (0.334)	1.401 (0.182)	1.393 (0.131)*	1.673 (0.408)	1.533 (0.282)
*PPMS* (*N* = *16*)	*DSC*	0.979 (0.008)*	0.997 (0.002)*	0.990 (0.005)*	0.932 (0.024)*	0.965 (0.005)
	*MSD*	0.114 (0.046)	0.066 (0.034)*	0.080 (0.040)*	0.230 (0.078)*	0.133 (0.037)
	*HD*	1.600 (0.344)	1.383 (0.226)	1.345 (0.143)*	1.698 (0.405)*	1.468 (0.188)
*SPMS* (*N* = *15*)	*DSC*	0.981 (0.008)*	0.996 (0.003)*	0.992 (0.006)*	0.943 (0.022)*	0.963 (0.005)
	*MSD*	0.121 (0.036)*	0.086 (0.037)*	0.089 (0.039)*	0.202 (0.074)*	0.148 (0.026)
	*HD*	1.585 (0.213)	1.413 (0.129)*	1.428 (0.115)	1.677 (0.476)	1.540 (0.236)
*RRMS* (*N* = *20*)	*DSC*	0.978 (0.008)*	0.998 (0.002)*	0.993 (0.004)*	0.921 (0.036)*	0.967 (0.006)
	*MSD*	0.130 (0.034)*	0.083 (0.033)*	0.093 (0.030)*	0.269 (0.093)*	0.154 (0.037)
	*HD*	1.936 (0.610)*	1.429 (0.115)*	1.420 (0.127)*	1.981 (0.773)	1.588 (0.305)
*All* (*N* = *95*)	*DSC*	0.980 (0.009)*	0.997 (0.003)*	0.994 (0.005)*	0.928 (0.027)*	0.967 (0.006)
	*MSD*	0.121 (0.047)*	0.082 (0.044)*	0.088 (0.041)*	0.256 (0.091)*	0.145 (0.037)
	*HD*	1.717 (0.466)*	1.430 (0.211)*	1.411 (0.137)*	1.778 (0.523)*	1.565 (0.288)

The script * represents significant differences (paired t-test with p < 0.05) between a rater/iNLS versus the proposed method.

**Table 3:** Evaluation results for the GM segmentation, with the mean (std) Dice score coefficient (DSC), mean surface distance (MSD), Hausdorff distance (HD) (all with respect to the consensus).

		Rater 1	Rater 2	Rater 3	*iNLS*	Proposed
*Controls* (*N* = *25*)	*DSC*	0.894 (0.039)*	0.927 (0.022)*	0.931 (0.020)*	0.776 (0.049)*	0.826 (0.046)
	*MSD*	0.100 (0.035)	0.063 (0.031)*	0.067 (0.028)*	0.106 (0.030)*	0.089 (0.030)
	*HD*	1.637 (0.274)*	1.285 (0.128)*	1.315 (0.135)*	1.723 (0.420)*	1.444 (0.204)
*CIS* (*N* = *19*)	*DSC*	0.908 (0.026)*	0.930 (0.040)*	0.933 (0.021)*	0.789 (0.046)*	0.835 (0.047)
	*MSD*	0.104 (0.044)	0.065 (0.035)*	0.064 (0.029)*	0.104 (0.029)*	0.083 (0.033)
	*HD*	1.654 (0.285)*	1.360 (0.251)	1.307 (0.208)	1.766 (0.475)*	1.393 (0.207)
*PPMS* (*N* = *16*)	*DSC*	0.907 (0.030)*	0.905 (0.038)*	0.911 (0.033)*	0.735 (0.073)*	0.780 (0.056)
	*MSD*	0.057 (0.027)*	0.061 (0.027)*	0.061 (0.023)*	0.118 (0.043)*	0.091 (0.039)
	*HD*	1.320 (0.200)	1.294 (0.134)*	1.239 (0.088)*	1.920 (0.536)*	1.611 (0.517)
*SPMS* (*N* = *15*)	*DSC*	0.914 (0.032)*	0.894 (0.041)*	0.903 (0.035)*	0.745 (0.100)	0.735 (0.069)
	*MSD*	0.054 (0.019)*	0.055 (0.030)*	0.052 (0.024)*	0.091 (0.039)	0.107 (0.056)
	*HD*	1.339 (0.234)	0.930 (0.117)	1.198 (0.353)*	1.517 (0.388)	1.444 (0.503)
*RRMS* (*N* = *20*)	*DSC*	0.923 (0.017)*	0.925 (0.027)*	0.906 (0.026)*	0.754 (0.049)*	0.793 (0.069)
	*MSD*	0.065 (0.024)	0.063 (0.022)*	0.063 (0.029)*	0.088 (0.032)	0.078 (0.031)
	*HD*	1.327 (0.136)	1.322 (0.186)	1.213 (0.309)*	1.821 (0.578)	1.441 (0.340)
*All* (*N* = *95*)	*DSC*	0.908 (0.031)*	0.918 (0.035)*	0.919 (0.029)*	0.762 (0.065)*	0.799 (0.066)
	*MSD*	0.079 (0.038)	0.062 (0.029)*	0.062 (0.027)*	0.101 (0.035)*	0.089 (0.038)
	*HD*	1.475 (0.280)	1.306 (0.171)*	1.261 (0.233)*	1.753 (0.489)*	1.461 (0.357)

The script * represents significant differences (paired t-test with p < 0.05) between a rater/iNLS versus the proposed method.

**Table 4:** Results from splitting the patients’ data into two groups depending on the presence of visible lesions at the C2/C3 level, with the mean (std) Dice score coefficient (DSC), mean surface distance (MSD), Hausdorff distance (HD) (all with respect to the consensus).

		With visible lesions (N = 45)	Without visible lesions (N = 25)
*CSA*	*DSC*	0.965 (0.143)*	0.970 (0.005)*
	*MSD*	0.143 (0.036)	0.139 (0.035)
	*HD*	1.534 (0.291)	1.538 (0.196)
*GM*	*DSC*	0.762 (0.067)*	0.838 (0.039)*
	*MSD*	0.096 (0.045)*	0.076 (0.025)*
	*HD*	1.525 (0.472)	1.364 (0.176)

The script * represents significant differences (paired t-test with p < 0.05) between groups.

**Table 5:** Test-retest measurements obtained from C2-3 spinal cord level.

		Volume (*mm*^3^)	Area (*mm*^2^)	COV (%)	DSC
*CSA*	Manual	1323.6 (92.6)	88.2 (6.2)	0.8 (0.5)	0.965 (0.005)
	Proposed	1321.4 (89.9)	88.1 (6.0)	1.2 (0.9)	
*GM*	Manual	209.8 (20.8)	14.0 (1.4)	7.2 (1.9)	0.791 (0.041)
	Proposed	186.6 (21.2)	12.4 (1.4)	7.4 (4.9)	

Mean (std) for volume, area, coefficient of variation (COV) and Dice score coefficient (DSC).

## References

[b1] Amukotuwa S. A. & Cook M. J. (eds) Spinal disease: neoplastic, degenerative, and infective spinal cord diseases and spinal cord compression (Clinical Gate, 2015).

[b2] Losseff N. a. . Spinal cord atrophy and disability in multiple sclerosis. A new reproducible and sensitive MRI method with potential to monitor disease progression. Brain 119, 701–708 (1996). 8673483 10.1093/brain/119.3.701

[b3] Schlaeger R. . Spinal cord gray matter atrophy correlates with multiple sclerosis disability. Annals of Neurology 76, 568–580 (2014). 25087920 10.1002/ana.24241 PMC5316412

[b4] Schlaeger R. . Association between thoracic spinal cord gray matter atrophy and disability in multiple sclerosis. JAMA Neurology 72, 897–904 (2015). 26053119 10.1001/jamaneurol.2015.0993 PMC6002864

[b5] Yiannakas M. C. . Feasibility of grey matter and white matter segmentation of the upper cervical cord *in vivo*: A pilot study with application to magnetisation transfer measurements. NeuroImage 63, 1054–1059 (2012). 22850571 10.1016/j.neuroimage.2012.07.048

[b6] Kearney H., Miszkiel K., Yiannakas M., Ciccarelli O. & Miller D. A pilot mri study of white and grey matter involvement by multiple sclerosis spinal cord lesions. Multiple Sclerosis and Related Disorders 2, 103–108 (2013). 25877631 10.1016/j.msard.2012.09.005

[b7] Hickman S., Hadjiprocopis A., Coulon O., Miller D. & Barker G. Cervical spinal cord MTR histogram analysis in multiple sclerosis using a 3D acquisition and a B-spline active surface segmentation technique. Magnetic Resonance Imaging 22, 891–895 (2004). 15234459 10.1016/j.mri.2004.01.056

[b8] Tench C. R., Morgan P. S. & Constantinescu C. S. Measurement of cervical spinal cord cross-sectional area by mri using edge detection and partial volume correction. Journal of Magnetic Resonance Imaging 21, 197–203 (2005). 15723367 10.1002/jmri.20253

[b9] Zivadinov R. . Comparison of three different methods for measurement of cervical cord atrophy in multiple sclerosis. AJNR. American journal of neuroradiology 29, 319–325 (2008). 17974604 10.3174/ajnr.A0813 PMC8118969

[b10] Horsfield M. a. . Rapid semi-automatic segmentation of the spinal cord from magnetic resonance images: Application in multiple sclerosis. NeuroImage 50, 446–455 (2010). 20060481 10.1016/j.neuroimage.2009.12.121 PMC2830007

[b11] McIntosh C., Hamarneh G., Toom M. & Tam R. C. Spinal cord segmentation for volume estimation in healthy and multiple sclerosis subjects using crawlers and minimal paths. *Proceedings - 2011 1*^*st*^ *IEEE International Conference on Healthcare Informatics, Imaging and Systems Biology, HISB 2011* 25–31 (2011).

[b12] Bergo F., Franca M., Chevis C. & Cendes F. Spineseg: A segmentation and measurement tool for evaluation of spinal cord atrophy. In *Information Systems and Technologies (CISTI), 7th Iberian Conference on*, 1–4 (2012).

[b13] Chen M. . Automatic magnetic resonance spinal cord segmentation with topology constraints for variable fields of view. NeuroImage 83, 1051–1062 (2013). 23927903 10.1016/j.neuroimage.2013.07.060 PMC3823375

[b14] El Mendili M.-M. . Validation of a semiautomated spinal cord segmentation method. Journal of Magnetic Resonance Imaging 41, 454–459 (2015). 24436309 10.1002/jmri.24571

[b15] De Leener B., Taso M., Cohen-Adad J. & Callot V. Segmentation of the human spinal cord. Magnetic Resonance Materials in Physics, Biology and Medicine 29, 125–153 (2016). 10.1007/s10334-015-0507-2 26724926

[b16] Asman A. J., Bryan F. W., Smith S. a., Reich D. S. & Landman B. a. Groupwise multi-atlas segmentation of the spinal cord’s internal structure. Medical Image Analysis 18, 460–471 (2014). 24556080 10.1016/j.media.2014.01.003 PMC4009677

[b17] Blaiotta C., Freund P., Curt A., Cardoso M. J. & Ashburner J. A probabilistic framework to learn average shaped tissue templates and its application to spinal cord image segmentation. In *Proceedings of the 24th Annual Meeting of ISMRM, Singapore*, 1449 (ISMRM, 2016).

[b18] Prados F. . Fully automated grey and white matter segmentation of the cervical cord *in vivo*. In *Proceedings of the 24*^*th*^ *Annual Meeting of ISMRM, Singapore*, 1133 (ISMRM, 2016).

[b19] Datta E. . Gray matter segmentation of the spinal cord with active contours in mr images. NeuroImage – (2016). In press. 10.1016/j.neuroimage.2016.07.062 27495383

[b20] Dupont S. M. . Fully-integrated framework for the segmentation and registration of the spinal cord white and gray matter. NeuroImage – (2016). In press. 10.1016/j.neuroimage.2016.09.026 27663988

[b21] El Mendili M.-M. . Cervical spinal cord atrophy profile in adult smn1-linked sma. Plos-ONE 11, e0152439 (2016). 27089520 10.1371/journal.pone.0152439 PMC4835076

[b22] Cardoso M. J. . STEPS: Similarity and Truth Estimation for Propagated Segmentations and its application to hippocampal segmentation and brain parcelation. Medical Image Analysis 17, 671–684 (2013). 23510558 10.1016/j.media.2013.02.006

[b23] Ta V., Giraud R., Collins D. & Coupé P. Optimized PatchMatch for near real time and accurate label fusion. MICCAI, Part III. LNCS 8675, 105–112 (2014). 10.1007/978-3-319-10443-0_14 25320788

[b24] Prados F. . Multi-Contrast PatchMatch Algorithm for Multiple Sclerosis Lesion Detection. In ISBI - Longitudinal MS Lesion Segmentation Challenge, 1–2 (2015).

[b25] Giraud R. . An optimized patchmatch for multi-scale and multi-feature label fusion. NeuroImage 124, Part A, 770–782 (2016). 26244277 10.1016/j.neuroimage.2015.07.076

[b26] Barnes C., Shechtman E., Golman D. B. & Finkelstein A. The generalized patchmatch correspondence algorithm. ECCV, Part III. LNCS 6313, 29–43 (2010).

[b27] Wheeler-Kingshott C. a. & Cohen-Adad J. (eds) Quantitative MRI of the Spinal Cord (Academic Press, San Diego, 2014).

[b28] Clarkson M. J. . Comparison of phantom and registration scaling corrections using the ADNI cohort. NeuroImage 47, 1506–1513 (2009). 19477282 10.1016/j.neuroimage.2009.05.045 PMC2800076

[b29] Leung K. K., Ridgway G. R., Ourselin S. & Fox N. C. Consistent multi-time-point brain atrophy estimation from the boundary shift integral. NeuroImage 59, 3995–4005 (2012). 22056457 10.1016/j.neuroimage.2011.10.068

[b30] Ourselin S., Roche A., Subsol G., Pennec X. & Ayache N. Reconstructing a 3D structure from serial histological sections. Image and vision computing 19, 25–31 (2001).

[b31] Modat M. . Global image registration using a symmetric block-matching approach. Journal of Medical Imaging 1, 024003 (2014). 26158035 10.1117/1.JMI.1.2.024003 PMC4478989

[b32] Modat M. . Fast free-form deformation using graphics processing units. Computer methods and programs in biomedicine 98, 278–284 (2010). 19818524 10.1016/j.cmpb.2009.09.002

[b33] Kearney H. . Grey matter involvement by focal cervical spinal cord lesions is associated with progressive multiple sclerosis. Multiple Sclerosis 1–11 (2015). 26432854 10.1177/1352458515604905

[b34] De Leener B., Kadoury S. & Cohen-Adad J. Robust, accurate and fast automatic segmentation of the spinal cord. NeuroImage 98, 528–536 (2014). 24780696 10.1016/j.neuroimage.2014.04.051

[b35] Brosch T. . Deep 3d convolutional encoder networks with shortcuts for multiscale feature integration applied to multiple sclerosis lesion segmentation. IEEE Transactions on Medical Imaging 35, 1229–1239 (2016). 10.1109/TMI.2016.2528821 26886978

[b36] Asman A. J., Smith S. a., Reich D. S. & Landman B. a. Robust GM/WM segmentation of the spinal cord with iterative non-local statistical fusion. Lecture Notes in Computer Science (including subseries Lecture Notes in Artificial Intelligence and Lecture Notes in Bioinformatics) 8149 LNCS, 759–767 (2013). 10.1007/978-3-642-40811-3_95 PMC3918679 24505736

